# Regulation of Survival Motor Neuron Gene Expression by Calcium Signaling

**DOI:** 10.3390/ijms221910234

**Published:** 2021-09-23

**Authors:** Kwangman Choi, Ansook Yang, Jiyeon Baek, Hyejeong Jeong, Yura Kang, Woosun Baek, Joon-Chul Kim, Mingu Kang, Miri Choi, Youngwook Ham, Min-Jeong Son, Sang-Bae Han, Janghwan Kim, Jae-Hyuk Jang, Jong Seog Ahn, Haihong Shen, Sun-Hee Woo, Jong Heon Kim, Sungchan Cho

**Affiliations:** 1 Natural Medicine Research Center, Korea Research Institute of Bioscience and Biotechnology, Cheongju 28116, Korea; m84choi@naver.com (K.C.); asyang@kribb.re.kr (A.Y.); 94world@naver.com (J.B.); jhj2456@hanmail.net (H.J.); eytro8608@daum.net (M.K.); mirichoi@kribb.re.kr (M.C.); hyt5272@kribb.re.kr (Y.H.); 2 Department of Medical Biotechnology, SoonChunHyang University, Asan 31538, Korea; 3 College of Pharmacy, Chungbuk National University, Cheongju 28160, Korea; shan@chungbuk.ac.kr; 4 Department of Cancer Biomedical Science, Graduate School of Cancer Science and Policy, National Cancer Center, Goyang 10408, Korea; kangyura95@gmail.com (Y.K.); bws2955@gmail.com (W.B.); 5 Cancer Molecular Biology Branch, Division of Cancer Biology, Research Institute, National Cancer Center, Goyang 10408, Korea; 6 College of Pharmacy, Chungnam National University, Daejeon 34134, Korea; joon.ch.kim@gmail.com (J.-C.K.); go81825@gmail.com (M.-J.S.); 7 Department of Biomolecular Science, KRIBB School of Bioscience, Korea University of Science and Technology, Daejeon 34113, Korea; jangjh@kribb.re.kr (J.-H.J.); jsahn@kribb.re.kr (J.S.A.); 8 Stem Cell Research Center, Korea Research Institute of Bioscience and Biotechnology, Daejeon 34141, Korea; janghwan.kim@kribb.re.kr; 9 Anticancer Agent Research Center, Korea Research Institute of Bioscience and Biotechnology, Cheongju 28116, Korea; 10 Gwangju Institute of Science and Technology, School of life Sciences, Gwangju 61005, Korea; haihongshen@gist.ac.kr

**Keywords:** spinal muscular atrophy (SMA), neuromuscular disease, SMN, pre-mRNA splicing, splicing modulator, calcium signaling, brefeldin A (BFA)

## Abstract

Spinal muscular atrophy (SMA) is caused by homozygous *survival of motor neurons 1* (*SMN1*) gene deletion, leaving a duplicate gene, *SMN2*, as the sole source of SMN protein. However, a defect in SMN2 splicing, involving exon 7 skipping, results in a low level of functional SMN protein. Therefore, the upregulation of SMN protein expression from the *SMN2* gene is generally considered to be one of the best therapeutic strategies to treat SMA. Most of the SMA drug discovery is based on synthetic compounds, and very few natural compounds have been explored thus far. Here, we performed an unbiased mechanism-independent and image-based screen of a library of microbial metabolites in SMA fibroblasts using an SMN-specific immunoassay. In doing so, we identified brefeldin A (BFA), a well-known inhibitor of ER-Golgi protein trafficking, as a strong inducer of SMN protein. The profound increase in SMN protein was attributed to, in part, the rescue of the SMN2 pre-mRNA splicing defect. Intriguingly, BFA increased the intracellular calcium concentration, and the BFA-induced exon 7 inclusion of SMN2 splicing, was abrogated by the depletion of intracellular calcium and by the pharmacological inhibition of calcium/calmodulin-dependent kinases (CaMKs). Moreover, BFA considerably reduced the expression of Tra2-β and SRSF9 proteins in SMA fibroblasts and enhanced the binding of PSF and hnRNP M to an exonic splicing enhancer (ESE) of exon 7. Together, our results demonstrate a significant role for calcium and its signaling on the regulation of SMN splicing, probably through modulating the expression/activity of splicing factors.

## 1. Introduction

Spinal muscular atrophy (SMA) is a rare neuromuscular disease occurring in approximately 1 in 6000–10,000 live births and is the leading genetic cause of infant mortality [1,2,3]. SMA is characterized by motor neuron degeneration, resulting in progressive muscle weakness and atrophy [4]. SMA patients are classified into four clinical types (I-IV), based on the age of disease onset, clinical severity and motor functions [5]. SMA is an autosomal recessive monogenic disease, which is mostly caused by the homozygous deletion of the *survival of motor neuron 1* (*SMN1*) gene. The resulting degree of SMN deficiency is closely correlated with disease severity. SMN is a ubiquitously expressed protein that is essential for the viability of all eukaryotic cells and plays crucial roles in diverse RNA metabolisms [6,7]. SMN forms a stable multi-protein complex (the SMN complex) with a group of gemin proteins in eukaryotic cells, and its most well-characterized function is in the assembly of small nuclear ribonucleoproteins (snRNPs), the major subunits of the spliceosome [8,9,10,11]. Even though SMN deficiency manifests as a motor neuron disease, its molecular consequences are evident, in an SMA mouse model, as profound disruptions in RNA metabolism in all tested tissues [12,13].

There are two *SMN* genes in humans, *SMN1* and *SMN2*, both of which encode for the same open reading frame (ORF). Most of SMA patients have homozygous *SMN1* deletions and are sustained by one or more copies of *SMN2*. However, due to a C/T substitution at exon 7, the splicing of the SMN2 pre-mRNA causes frequent exon 7 skipping (~80%). This produces a C-terminally truncated version of SMN protein (SMNΔ7) that is extremely unstable and almost undetectable. Consequently, a much lower level of fully functional full-length SMN is produced from *SMN2* [14], which is not sufficient to protect SMA patients against disease development. Intriguingly, SMA patients have a variable number of *SMN2* genes ranging from 2 to 6 copies; the level of SMN protein generally depends on the *SMN2* copy number and is inversely correlated with SMA severity [15].

Exon 7 inclusion of SMN2 pre-mRNA is regulated by a complex interplay between various cis-acting RNA elements and trans-acting protein factors. There are at least three cis-acting RNA elements (ESEs, TSL and ISS-N1) on or around exon 7, and more than 20 trans-acting factors (including SRSF1, Tra2-β, SRSF9, hnRNP A1/A2, hnRNP G, hnRNP M, hnRNP Q, PSF, Sam68 etc.) that act positively or negatively for exon 7 inclusion. Some trans-acting factors can bind directly to one or more cis-acting elements, and the rest bind indirectly, through protein-protein interaction. The amounts, activities and subcellular localization of these proteins can affect the alternative splicing of SMN2 exon 7 and can be modulated by posttranslational modifications, particularly phosphorylation. However, the cellular signaling and physiological conditions that regulate SMN2 exon 7 splicing remain largely unknown.

From the viewpoint of therapeutics, upregulation of survival motor neuron (SMN) expression from *SMN2* is a promising strategy to treat SMA. Moreover, rescuing the SMN2 splicing defect is undoubtedly considered the best way to achieve this. At the end of 2016, an oligonucleotide-based SMA drug (Nusinersen), which acts as a splicing corrector, was approved by the US Food and Drug Administration (FDA) and is currently being used for all ages and types of patients with SMA. This modified antisense oligonucleotide binds to the intronic splicing silencer (ISS-N1) element in intron 7 and physically blocks the binding of hnRNP A1/A2, a negative regulatory factor of exon 7 inclusions, to this region. However, due to its limited efficacy, impractical high cost, invasive administration and side effects, there remains a high demand for a better drug with improved features. More recently, in May 2019, onasemnogene abeparvovec (Zolgensma) was approved by the FDA as a gene replacement therapy based on an adeno-associated virus vector delivery. According to the clinical outcome, a single intravenous injection of this drug improves muscle movement and function in patients with SMA Type I [16]. Additionally, orally bioavailable synthetic small molecule, risdiplam (also known as RG7916), which effectively corrects SMN2 splicing defect with high specificity, was approved in August 2020 by the FDA for clinical use [17]. By comparison, small molecules originating from natural resources remain poorly explored. For example, only a few polyphenolic compounds (curcumin, resveratrol and EGCG) and quassinoids from plant extracts have been identified to induce SMN protein expression by modulating SMN2 splicing [18,19,20]. Even though a non-negligible number of FDA-approved drugs are based on the therapeutic activity of secondary metabolites of microorganisms, none have been reported to be effective for SMA [21].

Here, we identified brefeldin A (BFA) as a chemical modulator of SMN2 alternative splicing, from a screen of a library of microbial metabolites. BFA enhanced the expression of the SMN protein in SMA fibroblast cells partly by increasing exon 7 inclusion in SMN2 splicing. Moreover, we found that the effect of BFA on SMN2 splicing was mediated mainly by calcium and its signaling and involved the alteration of the expression or RNA-binding activities of Tra2-β, SRSF9, PSF and hnRNP M.

## 2. Results

### 2.1. Identification of BFA as a Chemical Inducer of SMN Protein

**Figure 1:**
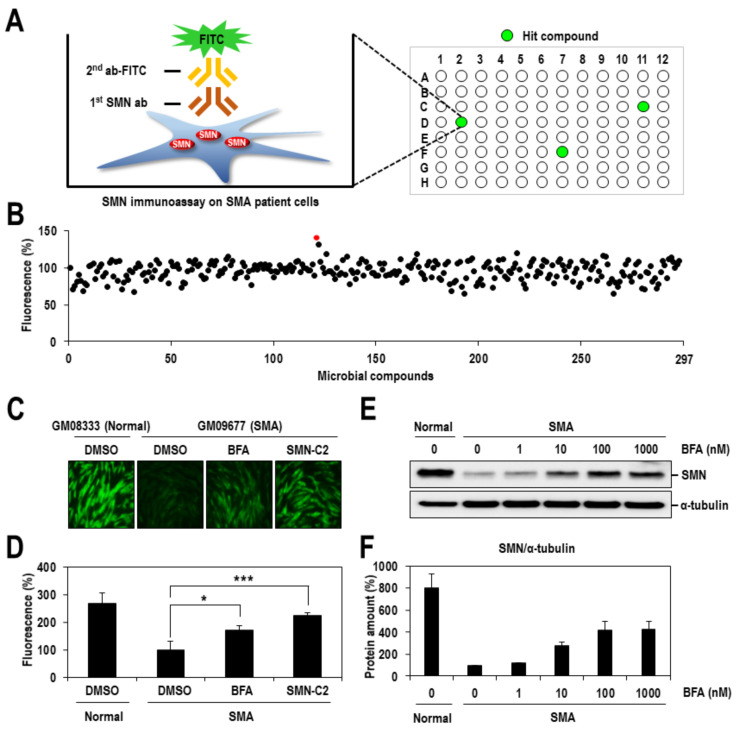
Identification of BFA as a potent inducer of SMN protein. (**A**) Schematic diagram of SMN immunoassay that was used in the screening. SMA fibroblasts (GM09677) were treated with microbial compounds (10 µM) for 24 h and stained with anti-SMN antibody and FITC-conjugated secondary antibody in 96well plate. (**B**) SMN signals in SMA fibroblasts were quantified using Cellomics ArrayScan instrument, and the activities relative to that from DMSO-treated SMA fibroblast were presented as a percentage. The result of BFA was indicated as a red-colored dot. (**C**) SMA fibroblasts were treated with DMSO, BFA (10 μM) or SMN-C2 (1 μM) for 24 h, stained with an anti-SMN antibody and FITC-conjugated secondary antibody and then visualized under an inverted fluorescence microscope. The color green indicates SMN-specific signal. Fibroblasts (GM08333) derived from a healthy individual were also included for comparison. (**D**) SMN signals in panel c were quantified using a Cellomics ArrayScan instrument and presented as a percentage of DMSO-treated SMA cells. The mean values and standard deviation were determined from three independent experiments. The level of statistical significance is labeled with an asterisk; * if *p* < 0.05 and *** if *p* < 0.001. (**E**) SMA fibroblasts were treated with a broad range of BFA concentrations, as indicated, for 24 h, and then cell lysates were harvested and subjected to Western blotting with an anti-SMN antibody. The amounts of α-tubulin were also analyzed as a loading control. (**F**) The amounts of SMN and α-tubulin proteins in panel e were quantified, and the amounts relative to those from DMSO-treated SMA fibroblasts were presented as a percentage. The mean values and standard deviation were determined from two independent experiments.

To identify any compound that increases SMN protein expression from microbial metabolites, we primarily established a robust assay system that enabled quantitative measurement of the amount of SMN protein in SMA fibroblasts. Previously, many researchers have devised various assay systems based on evaluating a single mechanism (e.g. SMN2 transcription, SMN2 pre-mRNA splicing or SMN/SMNΔ7 protein stability) in mammalian cells (e.g. 293T cells), mostly with the aid of reporter expression. In contrast, SMA fibroblasts (GM09677), which are more physiologically relevant cells derived from a patient with SMA Type I, were used in our immunoassay. SMN protein was detected through direct recognition by an SMN-specific antibody and subsequent signal amplification by fluorescein-conjugated secondary antibody (Figure 1A) and quantitatively measured using Cellnomics.

With this robust assay system, we conducted screening to identify any active compound from a microbial metabolite library composed of 297 compounds isolated from diverse bacterial and fungal strains [22]. SMA fibroblasts were treated with 10 µM of each various natural compounds for 24 h, and then SMN proteins were detected by immunoassay and quantified using Cellomics. The compound (#120), which was BFA, exhibited an appreciable increase in SMN signal by about 40% compared with the DMSO-treated control (Figure 1B). Furthermore, the positive effect of BFA on SMN signal was confirmed in a repeated experiment (Figure 1C), and the efficacy of BFA was lower than that of SMN-C2 (~100% of DMSO-treated control), which had been previously discovered as a potent SMN splicing modulator [23] (Figure 1D).

To further confirm the SMN-increasing activity of BFA and evaluate its potency, SMA fibroblasts were treated with increasing concentrations of BFA for 24 h and then analyzed using Western blotting with an anti-SMN antibody. BFA consistently exhibited strong SMN induction, reaching the maximum level, even at low concentrations (100 and 1000 nMs) (Figure 1E,F). Furthermore, its estimated potency for SMN induction was high with an EC_50_ of ~10 nM (Figure 1F). In contrast, α-tubulin was not affected under the same conditions. A similar result was obtained when another SMA fibroblast (GM00232) was treated with BFA (Figure S1). Collectively, these results indicated that BFA considerably enhanced the expression of SMN in SMA fibroblasts.

**Figure 2:**
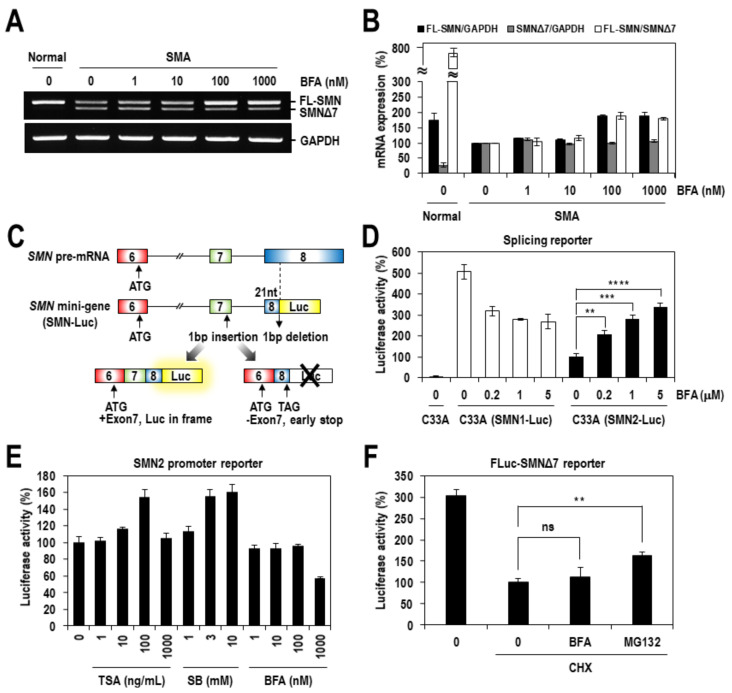
BFA rescues the SMN exon 7 splicing defect in SMA fibroblasts and SMN2-Luc splicing reporter cells. (**A**) Total RNAs were prepared from SMA fibroblasts treated with BFA as in Figure 1E and subjected to RT-PCR analysis for SMN2 exon 7 inclusion/exclusion. GAPDH mRNAs were also analyzed as a control. (**B**) The PCR products in panel a were quantified, and the intensities relative to those from DMSO-treated SMA fibroblasts are presented as a percentage. The mean values and standard deviation were determined from two independent experiments. (**C**) Schematic diagram of the *SMN* mini-gene splicing reporter. More details have been described in the Materials and Methods. (**D**) C33A cells stably expressing the SMN1- or SMN2-Luc splicing reporter were treated with indicated concentrations of BFA for 24 h, and then assayed for the firefly luciferase activity. The activity of each luciferase reporter was normalized to that from C33A-SMN2-Luc cells treated with DMSO and are presented as a percentage. The mean values and standard deviation were determined from three independent experiments. The level of statistical significance is labeled with an asterisk; ** for *p* < 0.01, *** for *p* < 0.001 and **** for *p* < 0.0001. (**E**) 293T cells were transfected with an SMN2 promoter reporter plasmid, treated with various concentrations of BFA for 24 h, and assayed for the firefly luciferase activity. Luciferase activities relative to that from DMSO-treated cells were presented as a percentage. Trichostatin A (TSA) and sodium butyrate (SB) were also tested as controls. The mean values and standard deviation were determined from two independent experiments. (**F**) 293T cells were transfected with plasmid expressing firefly lucfierase (FLuc)-fused SMNΔ7 protein, treated with BFA in the presence of cycloheximide (CHX) for 10 h, and assayed for the firefly luciferase activity. MG132 was also tested as a positive control. The mean values and standard deviation were determined from two independent experiments. The level of statistical significance is labeled with an asterisk; ns for not significant, ** for *p* < 0.01.

There may be three major ways that a chemical modulator induces the expression of SMN: transcriptional activation of the *SMN2* gene, splicing regulation of SMN2 pre-mRNA and regulation of SMN/SMNΔ7 protein stability. To define the molecular basis of SMN induction by BFA, an alteration in SMN2 alternative splicing was primarily analyzed. SMA fibroblasts were treated with increasing concentrations of BFA for 24 h, and then the total RNAs were prepared and subjected to RT-PCR to analyze SMN exon 7 inclusion/exclusion. The results showed that BFA dose-dependently increased the amount of exon 7-included mRNAs, whereas little appreciable alteration occurred in the amount of exon 7-excluded mRNAs (Figure 2A,B). This unequal change in the level of both mRNAs suggests that not only the transcription but also the alternative splicing might be involved in the effect of BFA. To confirm the effect of BFA on SMN2 alternative splicing, the luciferase-based SMN splicing reporter was employed (Figure 2C). In this assay, the increase in the firefly luciferase activity is tightly linked to increased exon 7 inclusions [24]. Indeed, a dose-dependent treatment of BFA gradually increased the firefly luciferase activity from the SMN2-Luc splicing reporter by more than three-fold at the highest concentration of 5 µM (Figure 2D). In contrast, no increase and even a decrease in the firefly luciferase activity was observed with the SMN1-Luc splicing reporter, likely due to the cell toxicity of BFA (Figure S2). Moreover, to define the molecular basis of BFA’s effect on SMN2 transcription, the luciferase-based SMN2 promoter reporter was constructed and used. Consistent with previous reports [25,26], the SMN2 promoter activity was increased by treating trichostatin A (TSA) or sodium butyrate (Figure 2E), which are well-known transcription-activating chemicals. In contrast, no increase in the SMN2 promoter activity was observed with BFA, suggesting that the transcriptional upregulation of the *SMN2* gene occurs through other than transcriptional initiation. The effect of BFA on SMNΔ7 protein instability was also examined using the luciferase-fused SMNΔ7 protein reporter, in which the firefly luciferase activity reflects the protein stability of SMNΔ7 [14]; however, we could not detect any positive effect (Figure 2F). Collectively, these results indicated that BFA induced the expression of the SMN protein by upregulating exon 7 inclusion in SMN2 pre-mRNA splicing and the transcription of the *SMN2* gene.

### 2.2. Calcium Mediates BFA-Induced SMN Expression Partly by Modulating the Alternative Splicing of SMN2 Exon 7

**Figure 3:**
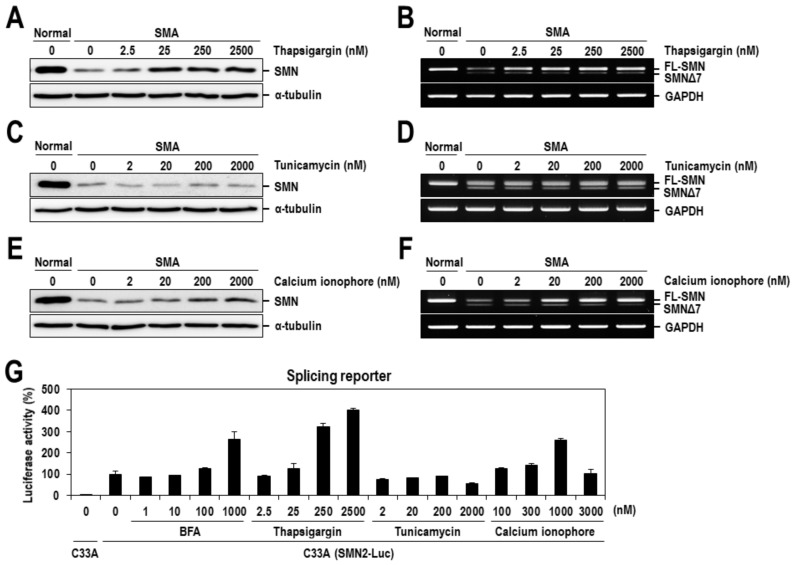
SMN2 exon 7 splicing is affected by calcium-modulatory chemicals. SMA fibroblasts were dose-dependently treated with thapsigargin (**A**), tunicamycin (**C**) or calcium ionophore (**E**) for 24 h and cell lysates were prepared and the expression of SMN protein was analyzed by Western blotting with anti-SMN antibody. Τhe α-tubulin was also analyzed as a loading control. (**B**,**D**,**F**) Total RNAs were prepared from the cells used in panel (**A**,**C**,**E**) and subjected to RT-PCR analysis for SMN2 exon 7 inclusion/exclusion. PCR products run on an agarose gel are presented. (**G**) SMN2-Luc cells (C33A) were treated with various concentrations of BFA, thapsigargin, tunicamycin or calcium ionophore for 24 h, and then assayed for the firefly luciferase activity. Luciferase activities were normalized to that from C33A(SMN2-Luc) cells treated with DMSO. The mean values and standard deviation were determined from three independent experiments.

BFA indirectly inhibits protein transport from the endoplasmic reticulum (ER) to the Golgi apparatus by preventing the formation of COPI-mediated transport vesicles [27,28,29], accumulating unfolded proteins inside the ER and leading to ER stress. Therefore, to determine whether ER stress is related to the SMN-inducing activity of BFA, two representative ER stress-inducing chemicals, thapsigargin and tunicamycin, were additionally tested on the SMN expression in SMA fibroblasts. Intriguingly, the level of SMN protein was considerably increased by dose-dependent treatment with thapsigargin (Figure 3A), an inhibitor of the calcium pump in the ER membrane, but not by tunicamycin, an inhibitor of glycosylation in the ER (Figure 3C). Concomitantly, treatment with thapsigargin increased the relative ratio of exon 7-included to exon 7-excluded mRNAs, similar to results obtained from the treatment with BFA (Figure 2A and Figure 3B); however, tunicamycin had no effect on the level of SMN2 mRNAs (Figure 3D). The ER stress-inducing mechanism of thapsigargin is directly related to an increase in cytosolic calcium levels by inhibiting calcium reuptake into the ER/sarcoplasmic reticulum (SR) [30], whereas that of tunicamycin is related to the inhibition of protein glycosylation [31]. Moreover, according to a recent report [32], short exposure to BFA transiently increases the level of cytosolic calcium in smooth muscle cells. These observations raised the possibility that BFA might act on the expression of SMN protein by modulating cytosolic calcium levels. Consistent with this possibility, we further observed that the calcium ionophore, a chemical inducing calcium influx and an increase in the level of cytosolic calcium, also increased the relative ratio of exon 7-included to exon 7-excluded mRNAs and the expression of SMN protein, similar to thapsigargin (Figure 3E,F). Moreover, the effect of calcium-related chemicals on SMN2 alternative splicing was also tested on the SMN2-Luc splicing reporter in C33A cells. As expected, the firefly luciferase activity, which tightly reflects an exon 7 inclusion event, was significantly increased by dose-dependent treatment with BFA, thapsigargin and calcium ionophore, but not by tunicamycin (Figure 3G), which agrees with their aforementioned effects on the expression of SMN mRNAs and protein (Figure 3A–F).

**Figure 4:**
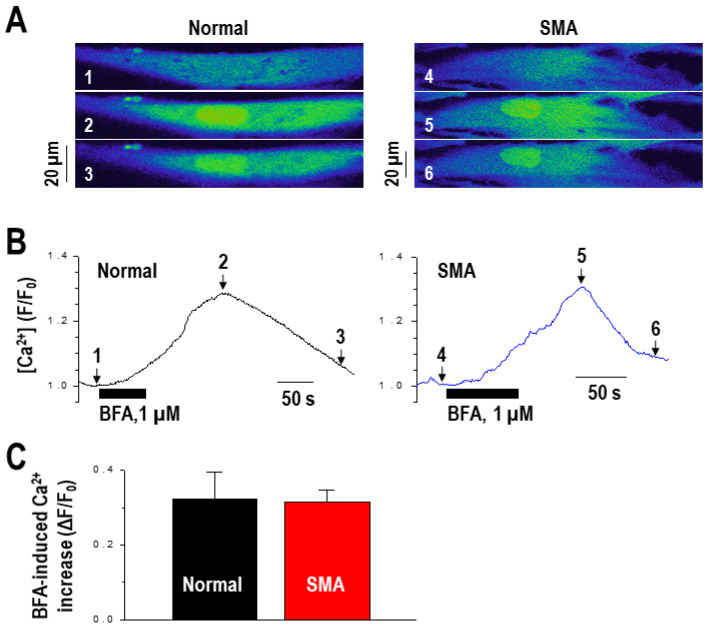
BFA increases the intracellular calcium concentration in normal and SMA fibroblasts. (**A**) Confocal fluo-4-Calcium images, taken in representative fibroblasts from normal and SMA at the time points marked in the panel, are shown. As the color scale of confocal images changes from blue to green and green to yellow tone, the fluorescence signal becomes higher. The images labeled with numbers (“1–6”) were selected at the time marked with the corresponding numbers in the signal traces in panel b. (**B**) Time course of calcium changes by BFA (1 µM), showing an increase in calcium level (F/F_0_). (**C**) Averaged calcium increases in normal (*n* = 10) and SMA (*n* = 24) fibroblasts were not significantly different (*p* > 0.05).

In order to further define the involvement of intracellular calcium in BFA-induced SMN expression, we investigated the effect of BFA on intracellular calcium levels in SMA fibroblasts. Normal and SMA fibroblasts were treated with 1 µM of BFA for 1–2 min, and intracellular calcium was measured using confocal imaging in combination with the calcium-sensitive fluorescent dye, fluo-4. Calcium signals were quantitatively measured as described in the Materials and Methods. Upon treatment, intracellular calcium levels noticeably increased in both normal and SMA fibroblasts (Figure 4A,B), and the magnitude of calcium increase was similar to that previously reported [32]. There was no significant difference between normal and SMA fibroblasts in the amplitude of calcium increase (Figure 4C).

**Figure 5:**
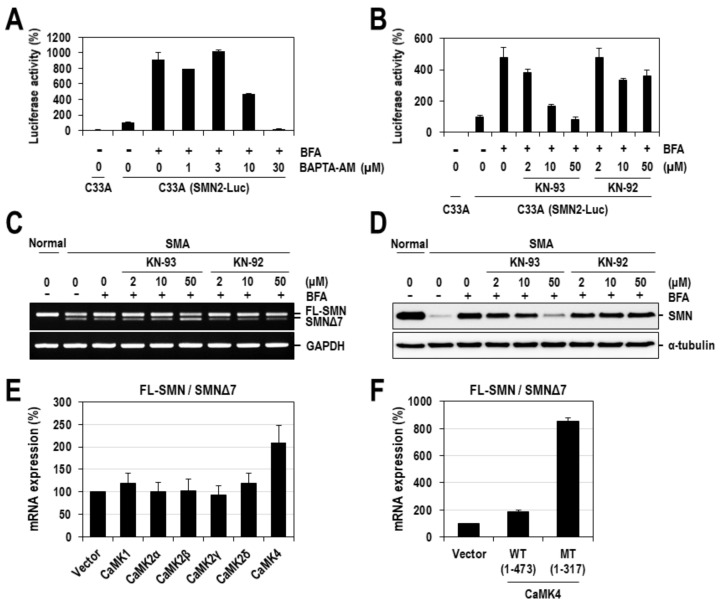
BFA-induced exon 7 inclusion is regulated by intracellular calcium and CaMKs. (**A**) Effect of BAPTA, a representative calcium chelator, on the alternative splicing of SMN2 exon 7 in C33A cells. SMN2-Luc cells were treated with various concentrations of BAPTA-AM in the presence of BFA (1 μM) for 24 h and then assayed for the firefly luciferase activity. Relative luciferase activities were calculated by normalizing to that from DMSO-treated cells and presented as a percentage. The mean values and standard deviation were determined from two independent experiments. (**B**) The effect of CaMK-specific inhibitor KN-93 and its inactive analog KN-92 on SMN2 splicing reporter cells. The mean values and standard deviation were determined from two independent experiments. (**C**) SMA fibroblasts were treated with the indicated concentrations of KN-93 or KN-92, along with BFA (1 μM) for 24 h, and then the total RNAs were prepared and subjected to RT-PCR for the analysis of exon 7 inclusion/exclusion of SMN2 mRNAs. GAPDH mRNAs were also analyzed as a control. (**D**) Lysates from cells used in panel c were harvested and subjected to Western blotting with anti-SMN antibody. α-tubulin was also analyzed as a loading control. (**E**) CaMK4 regulates the alternative splicing of SMN2 exon 7. The effect of CaMK4 on the SMN2-Luc splicing reporter. Various CaMKs (1, 2α, 2β, 2γ, 2δ or 4) were co-expressed with the SMN2-Luc splicing reporter in 293T cells for 48 h, and then total RNAs were prepared and subjected to RT-PCR analysis of exon 7 inclusion/exclusion. PCR products were quantified using ImageJ software, and the ratio of exon 7-included/-excluded (FL-SMN/SMNΔ7) mRNAs were calculated. Each value was normalized to the value from empty vector-transfected cells and presented as a percentage. The mean values and standard deviation were determined from three independent experiments. (**F**) Effect of a constitutively active form of CaMK4 on the SMN2-Luc splicing reporter. The experimental procedure was the same as in panel (**E**).

The involvement of calcium in the expression of SMN protein has been previously reported [33,34]. Since those studies have already defined the role of calcium in the transcriptional regulation of *SMN* genes, we herein focused on the aspect of splicing regulation. To ensure that BFA-induced exon 7 inclusion was mediated by calcium, we examined the effect of BAPTA, a representative calcium chelator, on SMN2 splicing. SMN2-Luc splicing reporter cells (C33A) were treated with various concentrations of BAPTA in the presence of BFA for 24 h and assayed for the firefly luciferase activity. As observed in Figure 2D and Figure 3G, treatment with BFA alone considerably increased the firefly luciferase activity. However, this stimulation of luciferase activity was gradually suppressed by co-treatment with BAPTA (Figure 5A). Intriguingly, luciferase activity, at the highest concentration of BAPTA (30 μM), decreased to levels below those in cells treated with neither BFA nor BAPTA. This overriding effect might be explained by the excessive chelation of endogenous calcium. A similar effect was also shown on SMN1-Luc splicing reporter in C33A cells (Figure S3). Next, we further examined if calcium/calmodulin-dependent protein kinases (CaMKs), whose stimulation is dependent on the increase in intracellular calcium, were involved in the effect of BFA on SMN2 exon 7 splicing. To examine this, we treated SMN2-Luc splicing reporter cells with a CaMK inhibitor KN-93 and its inactive analog KN-92, in the presence of BFA (1 µM). As expected, treatment with KN-93 dramatically suppressed the BFA-induced stimulation of the firefly luciferase activity, similar to the effect observed with BAPTA (Figure 5B). In contrast, KN-92, lacking CaMK-inhibitory activity, had a much weaker suppressive effect on SMN2 exon 7 splicing (Figure 5B). Similar results were observed in SMA fibroblasts as well (Figure 5C,D). BFA-induced exon 7 inclusion of SMN2 splicing was dose-dependently diminished by treatment with KN-93 but not with KN-92 (Figure 5C). Particularly, 50 µM of KN-93 completely abrogated the rescuing effect of BFA, reversing the splicing pattern to even surpass that of SMA cells. Consistent with SMN2 splicing, corresponding alterations were observed in the expression of SMN protein (Figure 5D). KN-93 but not KN-92 dose-dependently diminished the BFA-induced expression of SMN protein. Collectively, these results demonstrate a crucial role for calcium and CaMK(s) in BFA-induced exon 7 inclusion of SMN2 splicing.

### 2.3. CaMK4 Regulates the Alternative Splicing of SMN2 Exon 7

Next, we sought to determine which of the CaMKs contributed to SMN2 exon 7 alternative splicing. Considering that KN-93 is known as an inhibitor of CaMK1, 2 and 4 [35], we co-expressed each of the CaMKs (1, 2α, 2β, 2γ, 2δ and 4) with an SMN2-Luc splicing reporter in 293T cells for 48 h. Then, the total RNAs were prepared and subjected to RT–PCR to analyze the splicing pattern of the SMN2-Luc reporter gene. We observed that overexpression of CaMK4 alone exhibited an appreciably enhanced ratio of exon 7-included/-excluded mRNAs by more than 2-fold compared to the negative control (empty vector) (Figure 5E). In order to further confirm the positive role of CaMK4 in SMN2 exon 7 splicing, we employed a constitutively active form of CaMK4 and compared its effect with that of the wild-type. The wild-type and a constitutively active mutant form (1-317) of CaMK4 were co-expressed with an SMN2-Luc splicing reporter in 293T cells, and their effects on SMN2-Luc splicing were analyzed by RT-PCR. Intriguingly, in cells overexpressing a constitutively active form (1-317) of CaMK4, the ratio of exon 7-included/-excluded mRNAs was dramatically increased by more than 8-fold relative to the negative control, which was much higher than that observed with wild-type (Figure 5F). Together, these results indicate a positive role for CaMK4 in the regulation of SMN2 exon 7 splicing and also suggest its role as a mediator of the effects induced by BFA.

### 2.4. BFA Altered the Expression or RNA-Binding Activity of Splicing Factors

**Figure 6:**
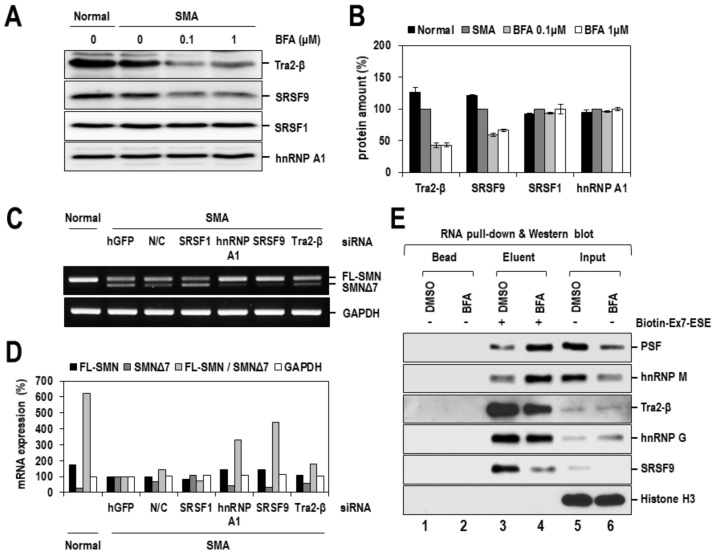
BFA reduces expression of SRSF9 and Tra2-β, and enhances SMN exon 7 ESE-binding activity of hnRNP M and PSF. (**A**) SMA fibroblasts were treated with BFA (0.1 and 1 µM) for 24 h, and total cell lysates were prepared and subjected to Western blotting with anti-SRSF1, -SRSF9, -Tra2-β and -hnRNP A1 antibodies. (**B**) The amounts of SRSF1, SRSF9, Tra2-β and hnRNP A1 proteins in panel a were quantified using ImageJ software. The relative amount of each protein was calculated by normalizing to the value from DMSO-treated SMA cells and presented as a percentage. The mean values and standard deviation were determined from two independent experiments. (**C**) The SMN2 exon 7 splicing effected by siRNA-mediated knockdown of splicing factors was analyzed by RT-PCR. siRNAs targeting GFP mRNA and scrambled siRNA were used as controls. GAPDH mRNA was also analyzed as a control. (**D**) The PCR products of FL-SMN and SMNΔ7 mRNAs in panel c were quantified using the ImageJ software. The relative amount of each PCR product was determined by normalizing to the value from GFP siRNA-transfected SMA cells, and are presented as a percentage. (**E**) 293T cells were treated with DMSO or BFA (0.1 μM) for 24 h, and the cell lysates were prepared and subjected to an RNA affinity assay. Cell lysates were incubated with biotinylated Exon 7-ESE RNAs, and ESE-bound proteins were precipitated by biotin-streptavidin bead interaction. Precipitated proteins were analyzed by Western blotting with anti-PSF, -hnRNP M, -Tra2-β, -hnRNP G and -SRSF9 antibodies. Histone H3 was also analyzed as a negative control.

According to previous reports, the alternative splicing of SMN exon 7 is regulated predominantly by the interplay of various trans-acting factors, such as SRSF1, Tra2-β, SRSF9, hnRNP A1/A2, hnRNP G, hnRNP M, hnRNP Q, PSF and Sam68, and cis-acting RNA elements on or around exon 7 [36,37]. Generally, relative protein amounts of splicing factors and their phosphorylation status are crucial for the regulation of alternative splicing. Thus, in order to define the underlying molecular basis of the effect of BFA on SMN2 exon 7 splicing, we first examined the expression of major trans-acting factors (SRSF1, Tra2-β, SRSF9 and hnRNP A1) after treating SMA fibroblasts with BFA for 24 h. We observed that the protein expression levels of Tra2-β and SRSF9 were significantly diminished by ~50%, compared to the DMSO control (Figure 6A,B). In contrast, the levels of SRSF1 and hnRNP A1 proteins were unchanged. Consistent with these results, siRNA-mediated knockdown of SRSF9 or Tra2-β, considerably enhanced the ratio of exon 7-included/-excluded SMN2 mRNAs (Figure 6C,D). These results suggest that BFA-induced exon 7 inclusion may occur, at least partly, through the modulation of the expression of splicing factors such as SRSF9 and Tra2-β. Moreover, we also investigated the possibility that the phosphorylation of major splicing factors, such as SR proteins, may be associated with the effect of BFA. The phosphorylation of SR proteins (e.g. SRSF1, SRSF3, SRSF4 and SRSF6) was monitored by Western blotting using the mAb1H4 antibody, which specifically recognizes phosphorylated SR peptides commonly existing in SR proteins [38]. Contrary to our expectation, there was no noticeable change in the levels of all phosphorylated SR proteins after treating SMA fibroblasts with BFA, which excluded the involvement of BFA-induced phosphorylation of SR proteins (Figure S4). Next, we investigated the binding of splicing factors to cis-acting elements. In particular, the exonic splicing enhancer (ESE), which is located in the middle of exon 7 and is known to be critical for exon 7 inclusion, was selected for this experiment. After treating 293T cells with 0.1 μM of BFA for 24 h, cell lysates were harvested, incubated with biotinylated Exon 7-ESE RNAs, and pulled down through biotin-streptavidin bead interaction. As previously reported [39], all tested proteins hnRNP M, PSF, SRSF9, Tra2-β and hnRNP G were shown to bind ESE RNAs, and their binding was considerably changed by treatment with BFA (Figure 6E). Intriguingly, despite the lowered expression of hnRNP M and PSF, their binding to Exon 7-ESE RNAs was enhanced in cells treated with BFA. The binding of Tra2-β and SRSF9 was decreased, likely due to their reduced expression. Collectively, these results suggested that the altered expression or RNA-binding activity of splicing factors, might contribute to BFA-induced exon 7 inclusion of SMN2 splicing.

## 3. Discussion

In this study, from a screen of 297 microbial compounds using a robust SMN-specific immunoassay, we identified a compound, BFA that significantly increased the expression of the SMN protein in SMA fibroblasts. Further analysis revealed that the SMN protein’s increase was partly due to the rescue of the SMN2 exon 7 splicing defect (Figure 2). To date, there is no report regarding the function of BFA on splicing regulation. Moreover, the effect of BFA on the splicing regulation of SMN2 exon 7 was potent, with an EC_50_ of ~50–200 nm (Figure 2A,D).

BFA is a macrocyclic lactone antibiotic produced from *Penicillium brefeldianum*, and its most well-studied function is the inhibition of the ER-Golgi transport of secretory proteins by targeting guanine nucleotide exchange factors (GEFs), such as GBF1 [40,41]. Inhibiting ER-Golgi trafficking causes the accumulation of unfolded or misfolded proteins inside the ER, leading to the occurrence of the unfolded protein response and ER stress [42,43]. Therefore, we examined the possibility that ER stress mediates the splicing regulatory function of BFA using two other prevalently used ER stress inducers, thapsigargin and tunicamycin. However, SMN2 splicing was affected only by thapsigargin. Given that these two chemicals have different modes of ER stress induction and that calcium is more crucial for the function of thapsigargin, we hypothesized that calcium was an important mediator in BFA’s splicing regulatory function. This hypothesis is supported by a recent report that BFA transiently induces calcium efflux from the ER and increases intracellular calcium levels [32].

Evidence obtained from our studies demonstrated the crucial role of calcium in BFA-regulated SMN2 splicing. First, treatment with a calcium ionophore, which facilitates the transport of calcium across the plasma membrane and induces the increase in cytosolic calcium concentration, exhibited a stimulatory effect on exon 7 inclusion in SMN2 splicing, leading to an enhanced expression of SMN protein in SMA fibroblasts (Figure 3E,F). Second, BFA increased the intracellular calcium concentration in SMA fibroblasts (Figure 4). Third, the depletion of intracellular free calcium, using BAPTA, completely abrogated the effect of BFA on SMN2 exon 7 inclusions (Figure 5A). Moreover, BAPTA alone induced exon 7 exclusion in the SMN1-Luc and SMN2-Luc splicing reporters (Figure S3); similar effects were also observed in SMA fibroblasts (Figure S5). Fourth, the pharmacological inhibition of CaMKs, which is activated by an increase in intracellular calcium concentration, significantly suppressed SMN2 exon 7 inclusions by BFA (Figure 5B–D). BFA-induced exon 7 inclusion of SMN2 splicing was completely abrogated by KN-93, a specific inhibitor of CaMK, but not by KN-92, its inactive analog, resulting in corresponding alterations in SMN protein amounts in SMA fibroblasts. Moreover, treatment with KN-93 alone had an appreciable effect on exon 7 exclusion (Figure S6). Fifth, CaMK4 regulates the alternative splicing of SMN2 exon 7 (Figure 5E). Notably, SMN2 exon 7 was completely included when a constitutively active form of CaMK4 was overexpressed in 293T cells (Figure 5F). Collectively, these results demonstrate the significant role of calcium and CaMK(s) in regulating SMN2 exon 7 splicing. Consistent with our results, associating calcium with SMN protein expression has been previously reported. In that study, high-throughput screening identified several calcium-modulatory chemicals (cardiac glycosides, monensin, calcium ionophore (A23187) and thapsigargin) as members of a primary hit class of compounds that increase the expression of SMN protein in SMA fibroblasts [44]. However, molecular details underlying the impact of calcium on SMN protein expression remained largely unexplored. Our study substantially improves the understanding of calcium-associated SMN regulation by revealing a significant role for calcium and its signaling in SMN splicing. In addition to the role of calcium in SMN splicing, calcium-dependent regulation of SMN expression at levels other than splicing may occur. According to a few studies, in spinal cord explant cultures from SMA mouse models, the N-methyl-D-aspartate (NMDA) receptor activation significantly enhanced SMN protein expression by controlling its transcription [33,45,46]. In this process, intracellular calcium was elevated, and CaMK2 was activated. Treatments with the CaMK inhibitor, KN-93, abolished the NMDA-mediated increase in SMN protein levels. Altogether, calcium and its signaling may regulate the protein expression of SMN by controlling its splicing and transcription.

There are several previous reports concerning the calcium-dependent splicing regulation. In this process, calcium-dependent activation of CaMKs and calcineurin plays an important role by phosphorylating or dephosphorylating several splicing factors, such as SR proteins and hnRNPs, which consequently relay an upstream stimulus to the splicing machinery. One of the well-studied examples is the calcium-dependent alternative splicing of the calcium-activated potassium channel, *slo*. In this process, CaMK4 is activated by calcium and phosphorylates hnRNP L, which in turn suppresses the inclusion of the STREX (stress axis-regulated exon) [47]. Based on extensive analysis of various STREX exons, the authors of that study identified a consensus sequence (CACATNRTTAT) in the proximity of 5′ splice sites that hnRNP L binds. Intriguingly, SMN exon 7 also contains a similar sequence (CACATTCCTTAAAT) near the 3′ splice site of exon 7. However, contrary to our expectation, GFP-hnRNP L overexpression in SMA fibroblasts did not affect SMN2 exon 7 splicing (Figure S7). Similar results were also observed in another study [48]. Alternatively, the expression or RNA-binding activity of other trans-acting factors crucial for SMN splicing may be altered by calcium, CaMKs or both. Indeed, SFSF9 and Tra2-β expression in SMA fibroblasts was considerably diminished by BFA treatment, which agrees with the observation that their siRNA-mediated knockdown induced exon 7 inclusion in SMN splicing (Figure 6C,D). Moreover, the binding of trans-acting factors to the exonic splicing enhancer (ESE) on exon 7 was significantly affected by treatment with BFA. Primarily, hnRNP M and PSF were extraordinary in that their binding to ESE was enhanced despite lowered expression (Figure 6E). Since these two proteins have already been reported as positive regulators of SMN2 exon 7 splicing [49,50], CaMKs (especially CaMK4) may directly or indirectly phosphorylate them, causing enhanced binding to ESE. According to a previous report, phosphorylating hnRNP M at S574 specifically controls the ability of the protein to inhibit intron removal of innate immune-activated transcripts, such as *IL-6* and *Mx-1*. PSF is phosphorylated by several kinases, such as MNK, NPM/ALK, BRK and GSK3-β, and its RNA-binding activity, subcellular localization or both are altered by phosphorylation. Hyperphosphorylation on multiple serine and threonine residues of PSF occurs during apoptosis, which induces its relocalization in the nucleus [51]. MNK kinase phosphorylates PSF on a serine residue and increases the binding of PSF to the TNF-α mRNA [52]. Similarly, the oncogenic fusion tyrosine kinase, nucleophosmin/anaplastic lymphoma kinase (NPM/ALK), phosphorylates PSF on Y293, which is located near the RRM1 domain (residues 299–363) in the N-terminus and increases the ability of PSF to bind RNA in anaplastic large-cell lymphoma [53]. BRK kinase phosphorylates tyrosine residue(s) at the C-terminal end of PSF, promoting the cytoplasmic relocalization of PSF and abrogating its binding to polypyrimidine RNAs [54]. Similar phosphorylation of PSF/hnRNP M may occur during calcium-activated exon 7 inclusion in SMN2 splicing. A more precise elucidation of the mechanism of CaMKs-dependent activation of hnRNP M and PSF will be pursued in future studies.

Our finding has been obtained mainly in SMA fibroblasts, which is derived from the skin that is pathophysiologically spared in patient. However, considering that the major pathologies and clinical manifestations of SMA are associated with the defect of motor neurons, further validation in more disease-relevant systems, such as induced pluripotent stem cell (iPSC)-derived SMA motor neurons, is required in future studies. iPSCs have been generated from SMA fibroblasts and further differentiated into motor neurons [55]. iPSC-derived SMA motor neurons have been extensively validated for many aspects of SMA and then widely used to examine the molecular basis of SMA pathologies and evaluate the pharmacological efficacy of drug candidates [56,57]. Notably, many reports show that SMN expression in iPSC-derived SMA motor neurons responds to pharmacological treatment similar to SMA fibroblasts [23,58,59].

**Figure 7:**
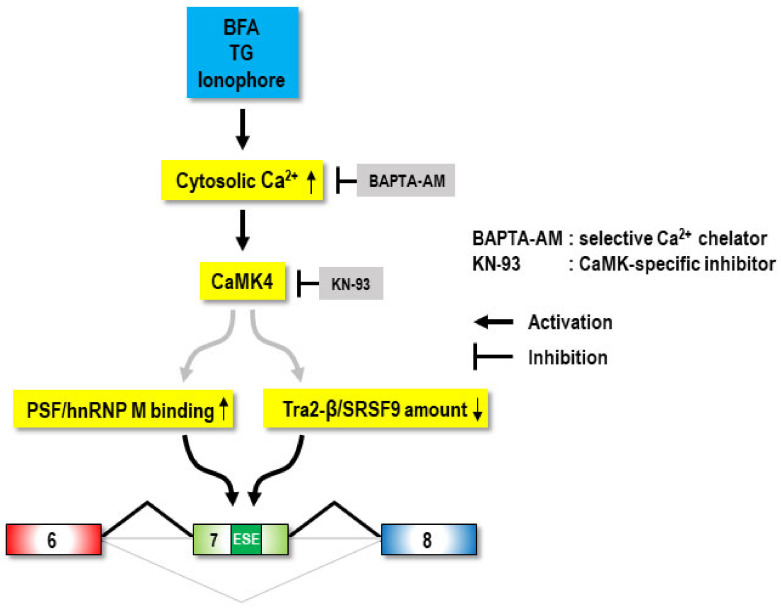
Expected pathway of calcium-mediated SMN splicing regulation. BFA, thapsigargin (TG) and calcium ionophore commonly increase the cytosolic calcium concentration, which is critical for the rescue of the SMN2 exon 7 splicing defect. In this pathway, CaMK4 may relay the stimulatory signal of the calcium to splicing factors, such as hnRNP M, PSF, Tra2-β and hnRNP A1, and consequently promote exon 7 inclusion in SMN splicing (black fold in the bottom). Thin and short arrows (upward and downward) indicate the increase or the decrease of RNA-binding or protein amount of trans-acting factors. The numbers (6, 7, and 8) indicate exon 6, 7, and 8 of SMN2 pre-mRNA, respectively.

This study identified a new function of the microbial metabolite, BFA, as a chemical modulator of SMN2 exon 7 splicing. Moreover, we revealed that BFA-induced rescue of the SMN2 exon 7 splicing defect was mediated by calcium and its signaling, which may require the activity of CaMK4, the reduced expression of SRSF9 and Tra2-β, and the enhanced ESE-binding activity of hnRNP M and PSF (Figure 7). Thus, our work identifies calcium signaling as a new axis for SMN splicing regulation and suggests its modulation as a therapeutic strategy for SMA.

## 4. Materials and Methods

### 4.1. Cell Cultures

Primary fibroblast cell lines derived from an SMA type I patient (GM09677 and GM00232) and an apparently healthy individual (GM08333) were purchased from Coriell Cell Repositories. They were maintained in Minimal Essential Medium (Gibco, Amarillo, TX, USA) containing 10% fetal bovine serum (Gibco), 1% L-glutamine (Gibco) and 1% penicillin-streptomycin (Gibco). 293T cells were purchased from Coriell Cell Repositories. They were maintained in Dulbecco’s Modified Eagle’s Medium (Hyclone, Logan, UT, USA) containing 10% fetal bovine serum (Welgene, Gyeongsan-si, Korea), 2 mM L-glutamine and 1% penicillin-streptomycin (Gibco). Stable cell lines expressing SMN1- and SMN2-Luc splicing reporter in C33A cells have been previously reported [24].

### 4.2. Plasmid Construction and DNA Transfection

SMN1- and SMN2-Luc plasmids were generated by Zhang et al. [24]. These plasmids contain the pre-mRNA of SMN1 or SMN2 from exon 6 to the 5′ part of exon 8 (21 nucleotides of exon 8), followed by the firefly luciferase gene (FLuc). To skip the in-frame translation termination codon (UAA), a cytosine was inserted between the 48th and 49th nucleotides in SMN exon 7. In addition, to prevent alternative translation initiation, the adenine in the start codon (AUG) was removed from the FLuc ORF. Consequently, exon 6-exon 7-exon 8-fused FLuc were expressed in-frame when exon 7 is included during SMN2 splicing. Stable SMN1-Luc and SMN2-Luc cells were generated by introducing SMN1 and SMN2 splicing minigene reporter plasmids, respectively, into C33A cells. In order to define which CaMK mediated the BFA-induced exon 7 inclusion of SMN2 splicing, several CaMK expression plasmids were constructed. For the generation of various N-terminal FLAG-tagged CaMKs (FLAG-CaMK1, 2α, 2β, 2γ, 2δ and 4), the ORFs of various human CaMKs were amplified from a human brain cDNA library (Clontech, Mountain View, CA, USA) with a designated oligomer set in Table S1. PCR products were inserted into Not I-digested pcDNA3-FLAG using the In-Fusion^®^ HD cloning kit (Takara Bio Inc. Kusatsu, Japan). The C-terminally truncated version of CaMK4 [CaMK4(1–317)] was amplified from the pcDNA3-FLAG-CaMK4(WT) with the following oligomers: sense, 5′-GATGACAAGGCGGCCGCGATGCTCAAAGTCACGGTGC-3′ and antisense, 5′-CTCGGATCCGCGGCCGCTCGAGTTAGAGCTTCTTTTGAGCGGTATC-3′. The PCR product was inserted into Not I-digested pcDNA3-FLAG using the In-Fusion^®^ HD cloning kit. For the generation of pcDNA3-FLAG-PSF, PCR was carried out with the human brain cDNA library and the following oligomers: sense, 5′-AGGCGGCCGCGGATCCCATGTCTCGGGATCGGTTC-3′ and antisense, 5′-TACCGAGCTCGGATCCTGCAGGAATTCGATATCAG-3′. The PCR product was inserted into BamH I-digested pcDNA3-FLAG using the In-Fusion^®^ HD cloning kit. For the generation of pcDNA3-FLAG-hnRNP M, PCR was carried out with the human brain cDNA library and the following oligomers: sense, 5′-AGGCGGCCGCGGATCCAATGGCGGCAGGGGTCGAAG-3′ and antisense, 5′-TACCGAGCTCGGATCCTGCTTAAGCGTTTCTATCAATTCG-3′. The PCR product was inserted into Not I-digested pcDNA3-FLAG using the In-Fusion^®^ HD cloning kit. All oligomers were purchased from Macrogen (Seoul, Korea) or Bionics (Seoul, Korea). All constructs were verified by DNA sequencing (CosmoGenetech, Seoul, Korea). For the transient expression of CaMKs, 293T cells were seeded at 2 × 10^5^ cells per well in 6-well plate and incubated overnight. Plasmids at 2 µg (0.1 µg of SMN2-Luc splicing reporter plasmid and 1.9 µg of CaMK expression plasmid) were then transfected using X-tremeGENE siRNA Transfection Reagent (Roche, Basel, Switzerland) for 48 h. In order to examine the effect of trans-acting factors on SMN2 splicing, siRNAs targeting mRNAs of SRSF1, SRSF9, hnRNP A1 and Tra2-β were purchased from Bioneer (Daejeon, Korea), and transiently transfected into SMA fibroblasts using the Lipofectamine RNAiMAX transfection reagent (Invitrogen, Waltham, MA, USA) according to the manufacturer’s protocol.

### 4.3. Antibodies and Chemicals

The mouse monoclonal anti-SMN (62E7) antibody was kindly provided by Gideon Dreyfuss (University of Pennsylvania, Philadelphia, PA, USA). Mouse monoclonal anti-SMN (Cat no. 610647, BD Transduction Laboratories, Franklin Lakes, NJ, USA), mouse monoclonal anti-α-tubulin (Sigma-Aldrich, Burlington, MA, USA) and Alexa Fluor 488-conjugated goat secondary antibody against mouse IgG (Invitrogen, Waltham, MA, USA) were used for immunostaining and Western blotting. Antibodies against SRSF9 (Cat no. sc-293314, Santa Cruz Biotech, Santa Cruz, CA, USA), SRSF1 (Cat no. sc-73026, Santa Cruz Biotech, Santa Cruz, CA, USA), Tra2-β (Cat no. sc-67117, Santa Cruz Biotech, Santa Cruz, CA, USA), hnRNP A1 (Cat no. sc-32301, Santa Cruz Biotech, Santa Cruz, CA, USA), PSF (Cat no. sc-271796, Santa Cruz Biotech, Santa Cruz, CA, USA), hnRNP M (Cat no. sc-20002, Santa Cruz Biotech, Santa Cruz, CA, USA), hnRNP G (Cat no. #14794, Cell Signaling Technology, Beverly, MA, USA), SC35 (mouse monoclonal, GTX11826, GeneTex, Irvine, CA, USA ) and histone H3 (Cat no. 06-599, Merck Millipore, Burlington, MA, USA) were used. Microbial metabolites were collected in-house from diverse strains of bacteria and fungi and used in the screening. The proteasome inhibitor MG132 (Calbiochem, San Diego, CA, USA), KN-93/KN-92 (Cayman Chemical Company, Ann Arbor, MI, USA), thapsigargin (Calbiochem), calcium ionophore/tunicamycin (Sigma-Aldrich, Burlington, MA, USA) were used in this study.

### 4.4. Immunoassay-Based Screening

SMA patient-derived fibroblasts (GM09677) were seeded at 8 × 10^3^ cells per well in black-well clear bottom 96-well plates (Griner). After 12 h, the cells were treated with 10 µM of each of the 297 microbial metabolites for 24 h. Cells were washed with 1 × PBS, fixed with 4% (*v*/*v*) paraformaldehyde in PBS for 3 min, permeabilized with 0.5% (*v*/*v*) Triton X-100 and 1% (*w*/*v*) bovine serum albumin (BSA) in PBS for 30 min, and incubated for 2 h in a blocking buffer containing mouse monoclonal antibody against SMN (BD Transduction Laboratories). The cells were then washed with PBS and incubated in blocking buffer containing Alexa Fluor 488-conjugated goat secondary antibody against mouse IgG (Invitrogen, Waltham, MA, USA) for 1 h. The cells were washed with PBS, stained with Hoechst 33342 dye (Invitrogen, Waltham, MA, USA) and analyzed with Cellomics (Thermo fisher Scientific, Waltham, MA, USA). The cell images were processed with neuronal profiling Ver. 3.5 (NPv3.5) BioApplication. ingle cells were identified using the Hoechst staining, and the fluorescent signals from the entire cell were detected and quantitatively measured. At least 300 cells under four fields of view were analyzed.

### 4.5. Quantitative Western Blot Analysis

The cell extracts were subjected to Western blot analysis with the appropriate antibodies as described previously [20]. Signals were detected using a WEST-ZOL plus Western Blotting Detection System (iNtRON Biotechnology, Seongnam-si, Korea) on a LAS-4000 Image analyzer (Fujifilm, Tokyo, Japan), and then quantitatively analyzed using the image analysis software ImageJ (NIH, Bethesda, MA, USA).

### 4.6. Analysis of the Alternative Splicing of SMN2 Exon 7

In order to analyze the alternative splicing of SMN2 exon 7, SMN1- or SMN2-Luc stable cells (C33A) were used as described previously [20]. Briefly, cells were seeded at 1 × 10^4^ cells per well in 96-well white plates and incubated overnight. Cells were treated with compounds for 24 h, and then luciferase activity was measured using the One-Glo Luciferase Assay System (Promega, Madison, WI, USA).

### 4.7. RT-PCR Analysis

For the analysis of SMN2 exon 7 splicing in SMA fibroblasts, total RNAs were isolated from cells using the TRIzol reagent (Invitrogen). First-strand cDNA was synthesized using the OmniScript RT kit (Qiagen, Hilden, Germany), which was primed with oligo-dT primers. The PCRs were performed using GoTaq Green master mix (Promega) and the following primers: SMN2 forward primer (5′-GGAAAGCCAGGTCTAAAATTCAA-3′) and SMN2 reverse primer (5′-CTATAACGCTTCACATTCCAGAT-3′) for the amplification of endogenous SMN2 mRNAs, SMN2-Luc reporter forward primer (5′-CTTGATGCTGATGCTTTGGG-3′) and SMN2-Luc reverse primer (5′-AAGCAATTGTTCCAGGAACC-3′) for the amplification of mRNAs from SMN2-Luc reporter, and the GAPDH forward primer (5′-AGAAGGCTGGGGCTCATTTG-3′) and GAPDH reverse primer (5′-AGGGGCCATCCACAGTCTTC-3′) for GAPDH mRNAs. The PCR cycling conditions were set as follows: initial denaturation at 95 °C for 30 s, 35 cycles of 30 s at 95 °C, 30 s at 55 °C, 60 s at 72 °C and a final extension time of 5 min at 72 °C. PCR products were resolved by electrophoresis using 2% agarose gels and visualized by RedSafe Nucleic Acid Staining solution (iNtRON). The band intensities were quantitated using ImageJ (NIH).

### 4.8. Real-Time PCR Analysis

The mRNA levels of FL-SMN, SMNΔ7 and GAPDH were quantified using TB Green^®^ Premix Ex Taq^TM^ II (Tli RNaseH Plus) Kit (TaKaRa, Kyoto, Japan) and the following primers: FL-SMN2 forward primer (5′-GCTCACATTCCTTAAATTAAGGAGAAA-3′), SMN2 Δ7 forward primer (5′-TGGCTATCATACTGGCTATTATATGGAA-3′) and SMN2 reverse primer (5′-TCCAGATCTGTCTGATCGTTTCTT-3′) for the amplification of endogenous FL-SMN and SMNΔ7 mRNAs, GAPDH forward primer (5′-CAACGGATTTGGTCGTATTGG-3′) and GAPDH reverse primer (5′-TGATGGCAACAATATCCACTTTACC-3′) for GAPDH mRNAs. Real-time PCR was carried out at the following temperatures for indicated times: Step 1: 48 °C (15 min); Step 2: 95 °C (10 min); Step 3: 95 °C (15 s); Step 4: 60 °C (1 min); Steps 3 and 4 were repeated for 40 cycles. The Ct values for each mRNA were converted to mRNA abundance using actual PCR efficiencies. The mRNA levels of FL-SMN and SMNΔ7 were primarily normalized to that of GAPDH, and relative amount of each mRNAs was presented as a percentage of DMSO-treated SMA fibroblasts.

### 4.9. Calcium Imaging

Calcium signal in cells was detected as previously described [60]. To measure the intracellular calcium signal, SMA fibroblasts were loaded with 3 μM of fluo-4 AM (Thermo Fisher Scientific) for 30 min. Dye-loaded cells in the cell chamber were continuously superfused with normal Tyrode’s solutions containing (in mM) 137 NaCl, 5.4 KCl, 10 HEPES, 1 MgCl_2_ and 10 glucose (pH 7.4). Calcium fluorescence was imaged at 1 Hz in 2-D using a laser scanning confocal imaging system (A1, Nikon, Tokyo, Japan) attached to an inverted microscope (Eclipse Ti, Nikon) fitted with a ×60 oil-immersion objective lens (Plan Apo, Numerical Aperture 1.4, Nikon). Dyes were excited at 488 nm using Ar laser (Ommichrome) and fluorescence emission was detected at >510 nm. The images were recorded by a workstation software, NIS Elements AR (v3.2, Nikon). In order to estimate calcium increases, the average resting fluorescence intensity (F_0_) was calculated from several frames immediately before the application of BFA. Tracings of calcium signals were shown as the average fluorescence of each cell normalized relative to the F_0_ (F/F_0_).

### 4.10. 5′-Biotinylated RNA Oligomer-Capture Pull-Down Assay

The sequence of the RNA oligomer for the RNA pull-down assay is 5′-Biotin-CAAAAAGAAGGAAGG-3′ (Ex7-ESE). The RNA oligomer was synthesized by Bioneer (Daejeon, Korea). 293T cells were treated with DMSO or BFA (0.1 µM) for 24 h, cell lysates were prepared with NE-PER™ Nuclear and Cytoplasmic Extraction Reagents (Thermo Fisher Scientific), and the nuclear fraction was subjected to an RNA affinity assay. Cell lysates were incubated with biotinylated Ex7-ESE RNAs, and ESE-bound proteins were precipitated by biotin-streptavidin bead interaction. Precipitated proteins were analyzed by Western blotting with antibodies against PSF (mouse monoclonal, clone D-8, sc-271796, Santa Cruz Biotech), hnRNP M (mouse monoclonal, clone 1D8, sc-20002, Santa Cruz Biotech), Tra2-β (Cat no. sc-67117, Santa Cruz Biotech), hnRNP G (rabbit monoclonal, clone D7C2V, #14794, Cell Signaling Technology) and SRSF9 (rabbit polyclonal, RN081PW, MBL). Histone H3 (rabbit monoclonal, clone D1H2, #4499, Cell Signaling Technology) was also analyzed as a negative control. As a secondary antibody, horseradish peroxidase-conjugated anti-rabbit (Vector Laboratories, Burlingame, CA, USA) and anti-mouse IgG (Vector Laboratories) were used. Enhanced chemiluminescence signals were detected and quantified by C-DIGIT (LI-COR) and/or X-ray film (AGFA) exposure.

## Data Availability

The data presented in this study are available on request from the corresponding author. The data are not publicly available due to privacy.

## Supplementary Materials

The following are available online at https://www.mdpi.com/article/10.3390/ijms221910234/s1.
