# Supplementary material for: Regulation of Survival Motor Neuron Gene Expression by Calcium Signaling
Source: Int J Mol Sci. 2021 Sep 23;22(19):10234. doi: 10.3390/ijms221910234 (PMC8508836; doi:10.3390/ijms221910234)
Supplement: Supplementary file 1 [file ijms-22-10234-s001.zip › ijms-1337919-supplementary.pdf]

Supplementary Data-Choi et al.

Regulation of Survival Motor Neuron Gene Expression by Calcium Signaling

Kwangman Choi [1,2,†], Ansook Yang [1,3,†], Jiyeon Baek [1,3], Hyejeong Jeong [1,3], Yura Kang [4,5], Woosun Baek [4,5], Joon-Chul Kim [6], Mingu Kang [1,3], Miri Choi [1,3], Youngwook Ham [1,7], Min-Jeong Son [6], Sang-Bae Han [3], Janghwan Kim [8], Jae-Hyuk Jang [7,9], Jong Seog Ahn [7,9], Haihong Shen [10], Sun-Hee Woo [6,*], Jong Heon Kim [4,5,*] and Sungchan Cho [1,7,*]

Supplementary Materials and Methods

Supplementary Table

Table S1. Oligonucleotides used in various FLAG-CaMK constructs.

S, sense; AS, antisense

Plasmid constructs	Sequence of oligonucleotides
pcDNA3-FLAG-CaMK1	S: 5′- GAT GAC AAG GCG GCC GCC ATG CTG GGG GCA GTG GAA G -3′<br>AS: 5′- CTC GGA TCC GCG GCC GCA GAG GAT CAT GAC CCG AGT C -3′
pcDNA3-FLAG-CaMK2α	S: 5′- GAT GAC AAG GCG GCC GCC AGG ATG GCC ACC ATC ACC -3′<br>AS: 5′- CTC GGA TCC GCG GCC GCC TGG TCC CTC AGT GGG GCA G -3′
pcDNA3-FLAG-CaMK2β	S:5′- GAT GAC AAG GCG GCC GCC ATG GCC ACC ACG GTG -3'<br>AS:5′- CTC GGA TCC GCG GCC GCG CAG CTC TTC ACT GCA GCG -3'
pcDNA3-FLAG-CaMK2γ	S: 5′- GAT GAC AAG GCG GCC GCG ATG GCT TCG ACC ACA ACC TGC -3'<br>AS: 5′- CTC GGA TCC GCG GCC GCC TTA GAT GTT TTG CCA CAA AGA GG -3'
pcDNA3-FLAG-CaMK2δ	S: 5′- GAT GAC AAG GCG GCC GCC AGC ATG GCC ACC ACC -3'<br>AS: 5′- CTC GGA TCC GCG GCC GCT GAG CTC ACT GCA GCG GTG C -3'
pcDNA3-FLAG-CaMK4	S: 5′- GAT GAC AAG GCG GCC GCG ATG CTC AAA GTC ACG GTG C-3'<br>AS: 5′- CTC GGA TCC GCG GCC GCT CGA GCT GTT TAG TAC TCT GGC AGG -3'

Supplementary Figures

Fig. S1

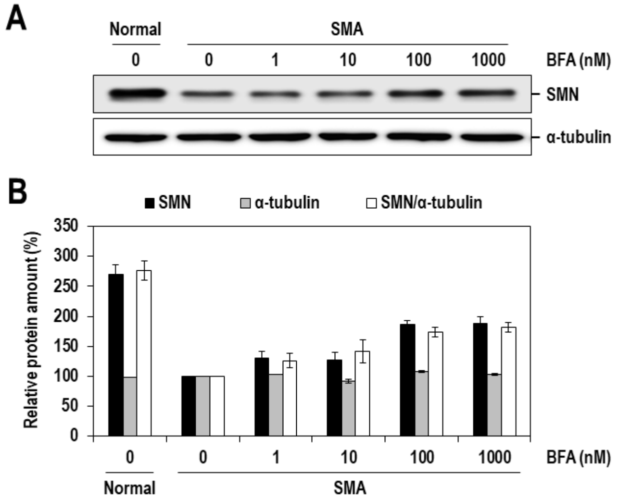

Fig. S1. The effect of BFA on SMN expression in SMA fibroblasts (A) SMA fibroblasts (GM00232) were treated with a broad range of BFA concentrations, as indicated, for 24 h, and then cell lysates were harvested and subjected to western blotting with an anti-SMN antibody. The amounts of α-tubulin were also analyzed as a loading control. **(B)** The amounts of SMN and α-tubulin proteins in panel a were quantified, and the amounts relative to those from DMSO-treated SMA fibroblasts were presented as a percentage. The mean values and standard deviation were determined from two independent experiments.

Fig. S2

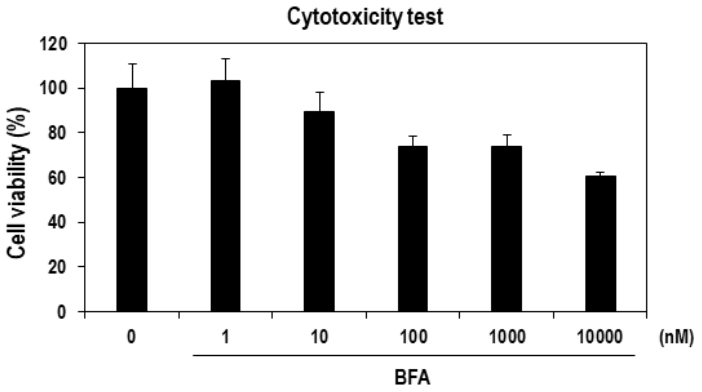

Fig. S2. The cytotoxic effect of BFA. SMA fibroblasts were treated with various concentrations of BFA for 24 h, and cell viability was measured by using CellTiter-glo reagent. Relative cell viability was presented as a percentage of DMSO-treated control. The mean values and standard deviation were determined from two independent experiments.

Fig. S3

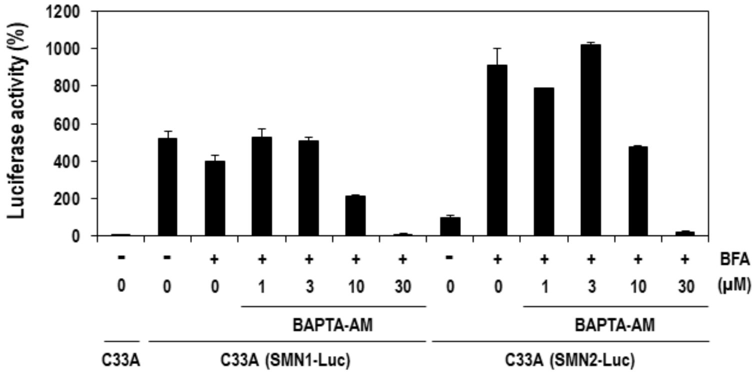

Fig. S3. The effect of BAPTA on the alternative splicing of SMN exon 7 in C33A cells. SMN1- or SMN2-Luc cells were treated with various concentrations of BAPTA-AM in the presence of BFA for 24 h and then assayed for the firefly luciferase activity. Relative luciferase activities were calculated by normalizing to that from DMSO-treated cells and presented as a percentage. The mean values and standard deviation were determined from two independent experiments.

Fig. S4

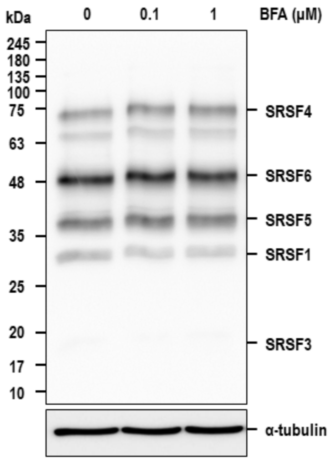

Fig. S4. The effect of BFA on the phosphorylation of SR proteins. Total protein extracts from 293T cells treated with BFA (0.1 and 1 μM) for 1 h were prepared and separated by SDS-PAGE, and phosphorylated SR proteins were monitored by western blotting using the phosphoSR monoclonal antibody (1H4). α-tubulin was analyzed as a loading control.

Fig. S5

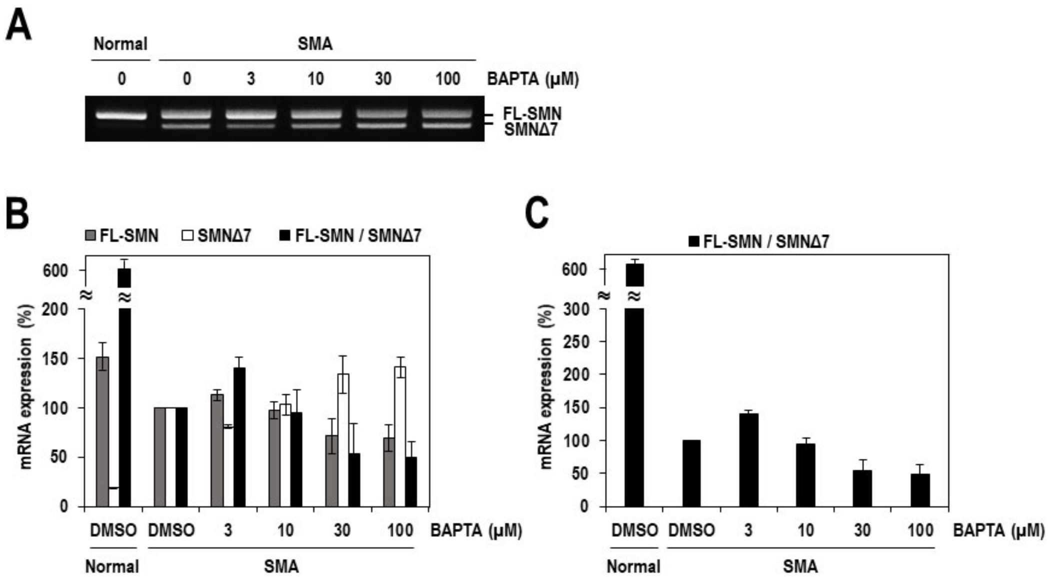

Fig. S5. The effect of BAPTA on the alternative splicing of SMN2 exon 7 in SMA fibroblasts. (A) SMA fibroblasts were treated with various concentrations of BAPTA-AM for 24 h. Total RNAs were prepared and subjected to RT-PCR for the analysis of SMN2 exon 7 inclusion/exclusion. The mean values and standard deviation were determined from two independent experiments. **(B)** DNA bands in panel a were quantified by using ImageJ software and relative amount of each mRNAs was presented as a percentage of DMSO-treated SMA fibroblasts. **(C)** The ratio of FL-SMN/SMNΔ7 mRNAs was determined from three independent experiments and shown a a percentage of DMSO-treated SMA fibroblasts.

Fig. S6

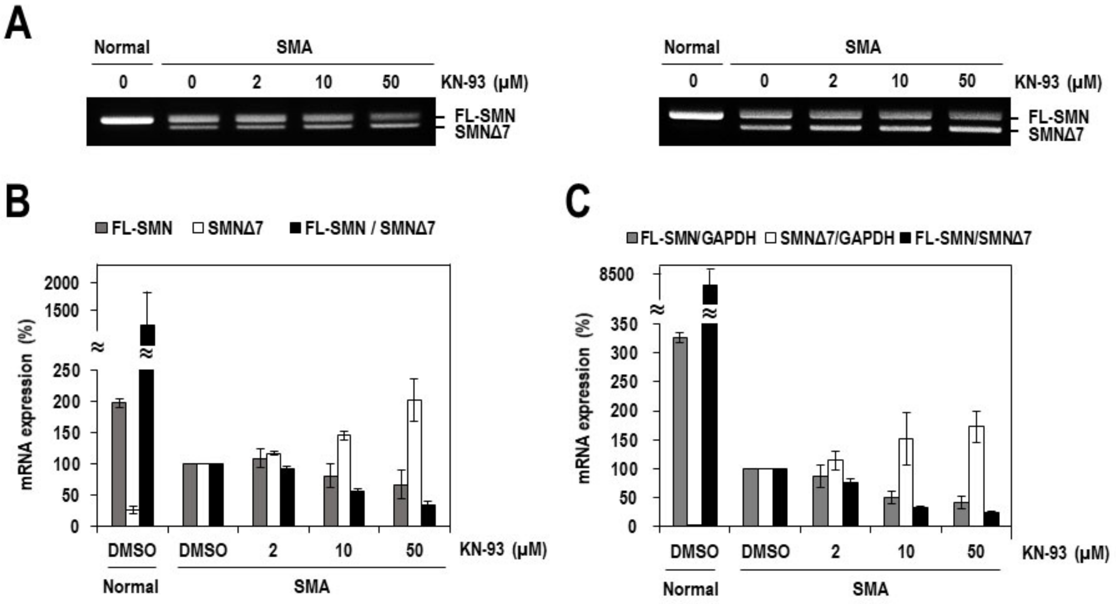

Fig. S6. The effect of KN-93 on the alternative splicing of SMN2 exon 7 in SMA fibroblasts. (A) SMA fibroblasts were treated with various concentrations of KN-93 for 24 h. Total RNAs were prepared and subjected to RT-PCR for the analysis of SMN2 exon 7 inclusion/exclusion. Two representative data were presented. **(B)** DNA bands in panel a were quantified by using ImageJ software, and relative amount of each mRNAs was presented as a percentage of DMSO-treated SMA fibroblasts. The mean values and standard deviation were determined from two independent experiments. **(C)** The mRNA levels of FL-SMN, SMNΔ7, and GAPDH in RNA samples used in RT-PCR were quantified using real-time PCR, and relative amount of each mRNAs was presented as a percentage of DMSO-treated SMA fibroblasts. The mean values and standard deviation were determined from two independent experiments.

Fig. S7

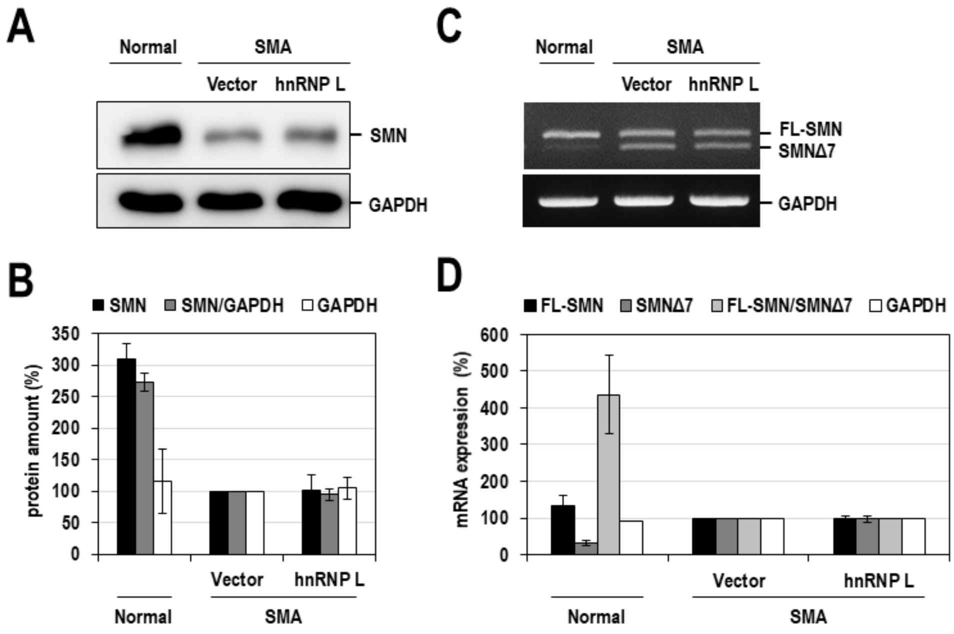

Fig. S7. The effect of hnRNP L on the alternative splicing of SMN2 exon 7 in SMA fibroblasts. (A) HnRNP L was overexpressed in SMA fibroblasts for 48 h, and total cell extracts were prepared and subjected to western blotting for the analysis of SMN protein. GAPDH proteins were also detected as a loading control. **(B)** The amount of SMN protein in panel a was quantified and presented as a percentage of vector-transfected SMA fibroblasts. The mean values and standard deviation were determined from two independent experiments. **(C)** Total RNAs were prepared from SMA fibroblast overexpressing hnRNP L, and subjected to RT-PCR for the analysis of SMN exon 7 inclusion/exclusion. GAPDH mRNAs were also detected as a control. **(D)** DNA bands in panel c were quantified and presented as a percentage of vector-transfected SMA fibroblasts. The mean values and standard deviation were determined from two independent experiments.
